# Supplementary material for: BRE/BRCC45 regulates CDC25A stability by recruiting USP7 in response to DNA damage
Source: Nat Commun. 2018 Feb 7;9:537. doi: 10.1038/s41467-018-03020-6 (PMC5803202; doi:10.1038/s41467-018-03020-6)
Supplement: Supplementary file 1 — Supplementary Information [file 41467_2018_3020_MOESM1_ESM.pdf]

Supplementary Methods

MSCV virus production and transduction to ES cells

To generate stable virus producing cells respective plasmids were transfected into packaging cell line GP+E86 (ATCC, CRL 9642 ™) and selected cells with appropriate antibiotics (Hygromycin or G418). Retroviral infections were performed in a 10 cm dish by co-culturing mouse ES cells ($1\text{-}2 \times 10^6$) with packaging cells (3×10^6) mitotically inactivated by mitomycinC (MMC) treatment (10 μg/ml for 2 hours), in the presence of 8 μg/ml polybrene (Santa Cruz Biotech) for 48 hours. The transduced cells were washed with PBS and then plated at lower density and selected for either in HAT media or for antibiotic resistance. Individual colonies were picked into 96 well plates and further analyzed either by Southern or Western blot.

Deletion of conditional *Brca2* or *Brca1* allele in ES cells

To delete the conditional allele, 20 μg Pgk-Cre plasmid was electroporated into ES cells and HAT resistant colonies were selected. Individual colonies were then analyzed by Southern hybridization for the loss of conditional allele as described previously [1,2].

Splinkerette PCR

Genomic DNA was isolated from *Brca2* null cells, digested with *Eco*RI, and ligated to the splinkerette linker overnight. Polymerase chain reaction (PCR) was performed on the ligation reaction using gene-specific (Cre or Hygro) primers and splinkerette-specific primers followed by a nested PCR performed using primers recognizing the long terminal repeat (LTR) of MSCV and the splinkerette linker. Products were separated on 1% agarose gels. Excised DNA bands were purified using MiniElute columns (Qiagen, Valencia, CA) and sequenced directly using the Big Dye Cycle Sequencing kit (Perkin Elmer, Shelton, CT) and an ABI Model 373A DNA Sequencer (Applied Biosystems, Foster City, CA).

Expression analysis

Total RNA was prepared using RNA-BEE (Tel-Test, Inc.) according to the manufacturer's protocol and cDNA samples were prepared from total RNA using SuperScript III first strand synthesis kit (Invitrogen). To quantitate expression levels a Brilliant II SYBR Green QPCR kit (Stratagene) was performed according to the manufacturer's instructions. GAPDH was used as internal control. Gene-specific primer sequences and thermo cycling conditions are available on request.

For WB analysis, proteins were extracted in RIPA buffer (50 mM Tris-HCl, pH 7.4, 1 mM EDTA, 150 mM NaCl, 0.1% SDS, 1% Triton X-100, 0.25% sodium deoxycholate, 1 mM sodium fluoride, 1 mM orthovanadate). Proteins were separated in Tris-Glycine, 4-12% Bis-Tris (Invitrogen) or 3-8% Tris- acetate (Invitrogen) gels by electrophoresis, and ECL Plus Western Blotting Detection system (Amersham) was used for chemiluminescent detection. Determining the band intensity of the samples normalized with the GAPDH input band performed to quantify Western blot. Band intensities were quantified using GeneTools software (Syngene).

Cell cycle analysis

For BrdU (5-bromo-2'-deoxy-uridine) staining, cells were irradiated with 6 Gy irradiation, incubated for 1hr and then pulse-labeled with 10 μM BrdU (Boehringer Mannheim, Mannheim, Germany) for 15 minutes. Cells were harvested and fixed in 70 % ethanol. Cells were then stained for both DNA content and BrdU incorporation by the acid denaturation-protease method by using fluorescein isothiocyanate (FITC)-conjugated anti-BrdU (Becton Dickinson) antibody. Cells were incubated with RNase-propidium iodide solution (BD biosciences) for 30 minutes before analyzing by flow cytometry.

For the transient G2/M transition checkpoint assay, asynchronous cells were exposed to 2 Gy IR and then incubated at 37°C for 8 h. Cells were fixed with 70% ethanol and stained with FITC-conjugated rabbit anti-phospho-histone H3 antibody (#9701S, Cell signaling). The stained cells were treated with RNase-propidium iodide solution (BD biosciences) for 30 min at room temperature and then analyzed by flow cytometry.

For radio-resistant DNA synthesis analysis log phase cells were treated with media containing 10 nCi/ml ^{14}C-thymidine overnight, cells were then washed and further incubated for 24 hrs. Cells were exposed to various dose of irradiation, incubated for 30 minutes and then pulsed 2.5 μCi /ml ^{3}H-thymidine for 15 minutes. Cells were harvested and fixed with 70% methanol. Fixed cells were adsorbed onto Whatman filter disks using vacuum manifold, washed, dried and counted using a liquid scintillation counter. The tritium counts were normalized against ^{14}C counts. DNA synthesis is expressed as percent of counts per minute in DNA of irradiated cells compared to unirradiated controls.

Drug and radiation sensitivity assay

For drug sensitivity assay to different drugs and ionizing radiation 10,000 ES cells per well were plated (20,000 cells per well for mutants to compensate for lower seeding and growth efficiency) in 96-well plates. The next day, cells were treated with M15 medium containing appropriate concentrations of drugs. For gamma-irradiation, plates were exposed to a ^{137}Cs source at 146.3 RAD min-1 without media change. After 48 hours of drug treatment, cell proliferation was measured by XTT (2,3-bis(2-methoxy-4-nitro-5-sulfophenyl)-5-[(phenylamino)carbonyl]-2H-tetrazolium hydroxide) assay as described previously [2].

For clonogenic survival assay 100,000 cells were seeded in 3.5 cm dish. The next day plates

were irradiated with media and incubated to form colonies. Colonies were stained with methylene blue [2% methylene blue (wt/vol) in 70% ethanol for 15 min followed by washing with 70% ethanol]. Colonies were quantified using ImageJ software.

All samples were analyzed in triplicate. For each cell lines two independent clones were checked and they behaved identically. The percentages of surviving cells were expressed as compared to untreated cells.

RAD51 foci formation assay

Cells were grown on lysine-coated coverslips and 5 hrs after 6 Gy IR cells were fixed with 4% paraformaldehyde for 5 min and permeabilized in PBS buffered 0.1% Triton X-100 for 10 min. Antibody staining was performed as described previously [3]. Nuclei were imaged using a Zeiss LSM 510 inverted confocal laser-scanning microscope equipped with a ZEISS Axiovert 100 microscope with a 63X immersion oil objective. Two independent clones were used for each genotype and they behaved identically.

Karyotyping

ES cells were treated with colcemid (Invitrogen) for 1.5 hr and karyotyping was performed as described previously [2]. For each genotype randomly selected 50 metaphase spreads with 40 chromosomes were counted for aberrations blindly. For each genotype two independent clones were analyzed.

A

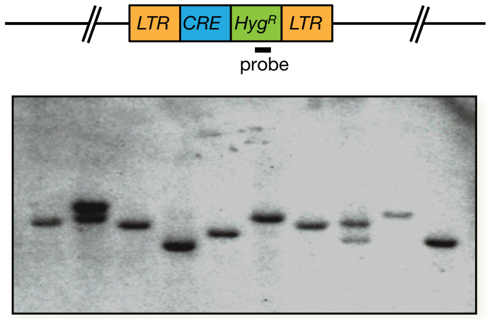
LTR
CRE
Hyg^R
LTR
probe

B

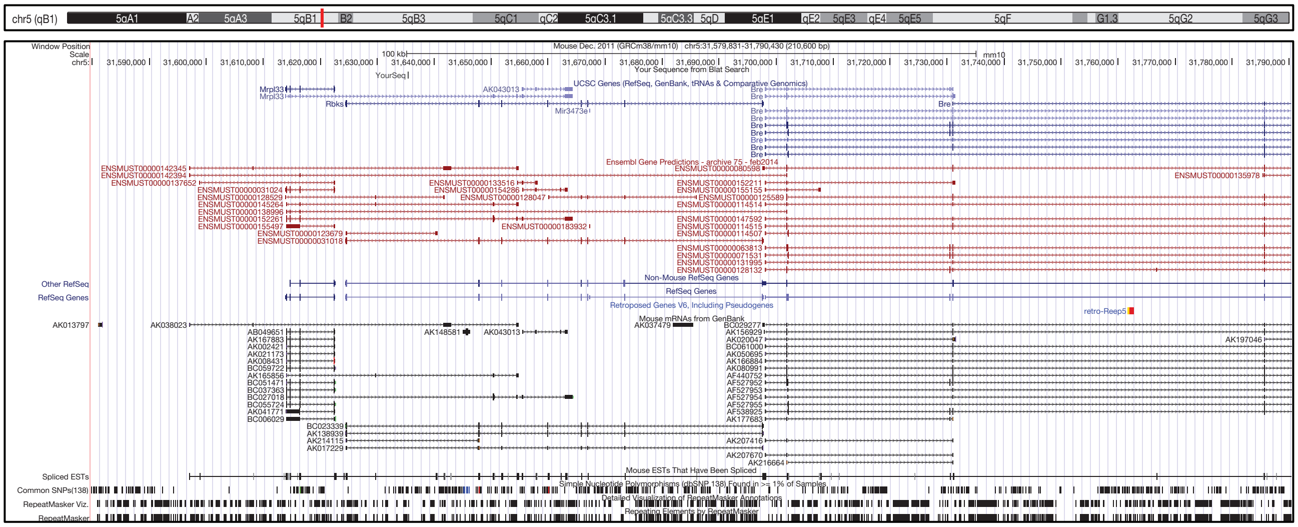
chr5 (qB1)
Window Position
Scale
chr5:
UCSC Genes (RefSeq, GenBank, tRNAs & Comparative Genomics)
Ensembl Gene Predictions - archive 75 - feb2014
Other RefSeq
RefSeq Genes
Non-Mouse RefSeq Genes
Retroposed Genes V6, Including Pseudogenes
Mouse mRNAs from GenBank
Spliced ESTs
Mouse ESTs That Have Been Spliced
Common SNPs(138)
RepeatMasker Viz.
RepeatMasker

C

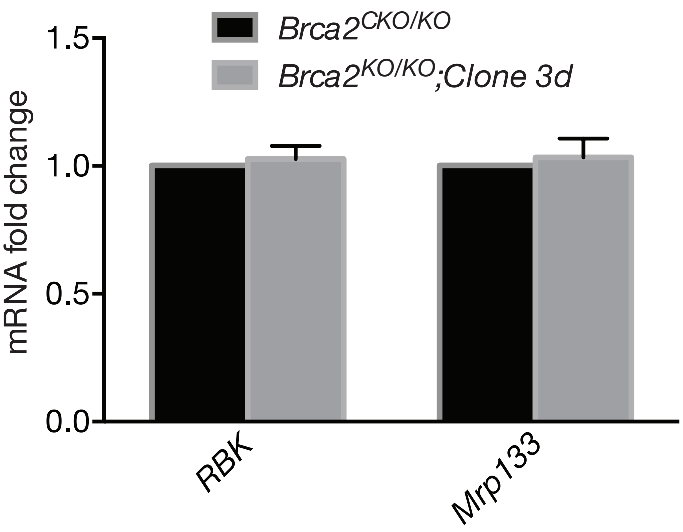
Brca2^CKO/KO
Brca2^KO/KO;Clone 3d
mRNA fold change
1.5
1.0
0.5
0.0
RBK
Mrp133

Supplementary Figure 1. Identification of BRE as a genetic interactor of BRCA2 using MSCV-based insertional mutagenesis screen. (**A**) Southern blot showing the integration of MSCV into mES cell genome. Top panel depicts the MSCV cassette used for integration. Thick line represents the probe used. (**B**) Viral insertion site identified in chromosome 5. A snapshot for BLAST results in UCSC genome browser (http://www.genome.ucsc.edu/) (**C**) Quantification of mRNA expression of *Rbks* and *Mrp133* in mES cells. Data are shown as mean ± s.d.

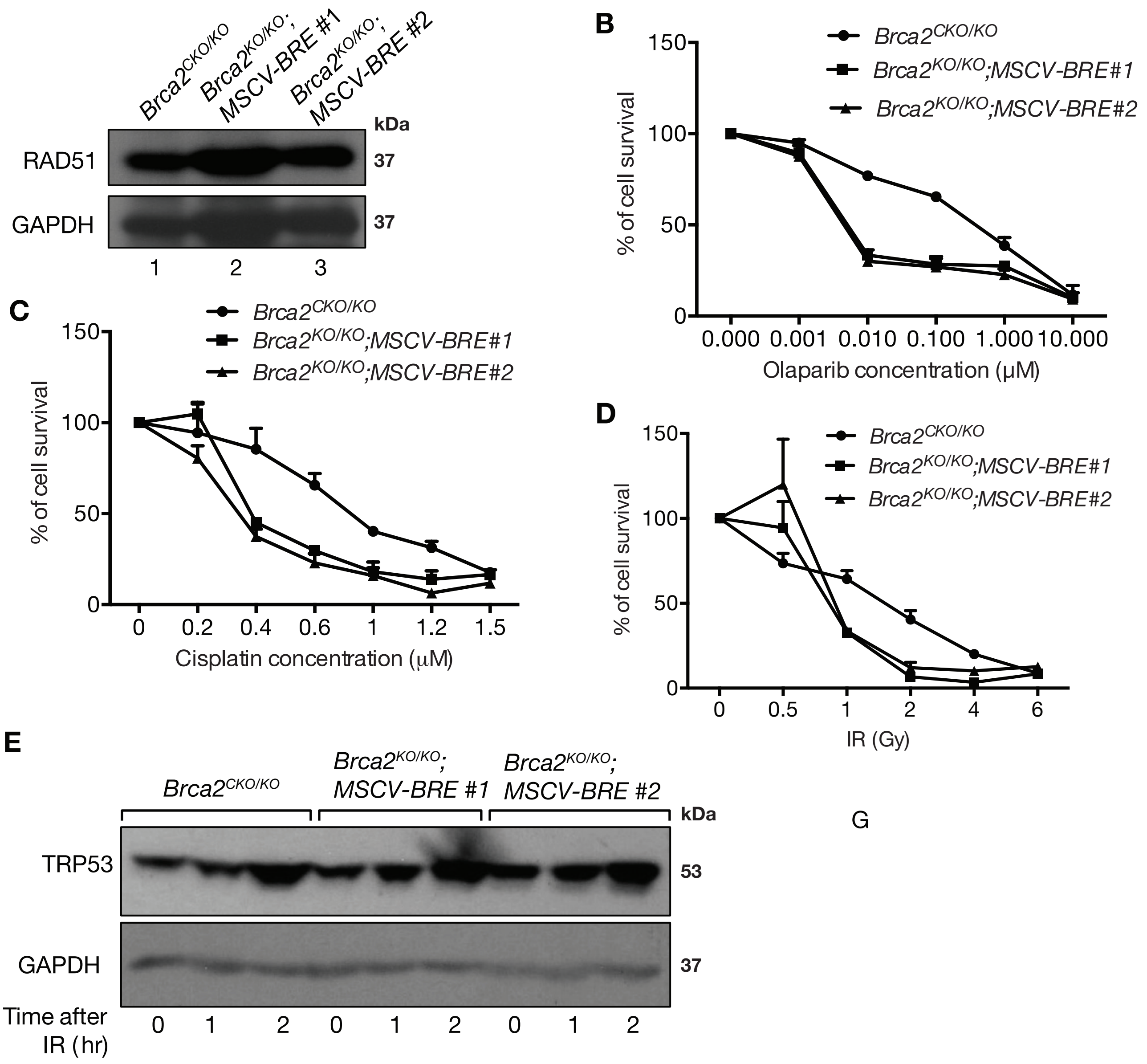
Brca2CKO/KO
Brca2KO/KO; MSCV-BRE #1
Brca2KO/KO; MSCV-BRE #2
kDa
RAD51
37
GAPDH
37
1 2 3
B
150
100
50
0
% of cell survival
Brca2CKO/KO
Brca2KO/KO;MSCV-BRE#1
Brca2KO/KO;MSCV-BRE#2
0.000 0.001 0.010 0.100 1.000 10.000
Olaparib concentration (μM)
C
150
100
50
0
% of cell survival
Brca2CKO/KO
Brca2KO/KO;MSCV-BRE#1
Brca2KO/KO;MSCV-BRE#2
0 0.2 0.4 0.6 1 1.2 1.5
Cisplatin concentration (μM)
D
150
100
50
0
% of cell survival
Brca2CKO/KO
Brca2KO/KO;MSCV-BRE#1
Brca2KO/KO;MSCV-BRE#2
0 0.5 1 2 4 6
IR (Gy)
E
Brca2CKO/KO
Brca2KO/KO; MSCV-BRE #1
Brca2KO/KO; MSCV-BRE #2
kDa
TRP53
53
GAPDH
37
Time after IR (hr)
0 1 2 0 1 2 0 1 2
G

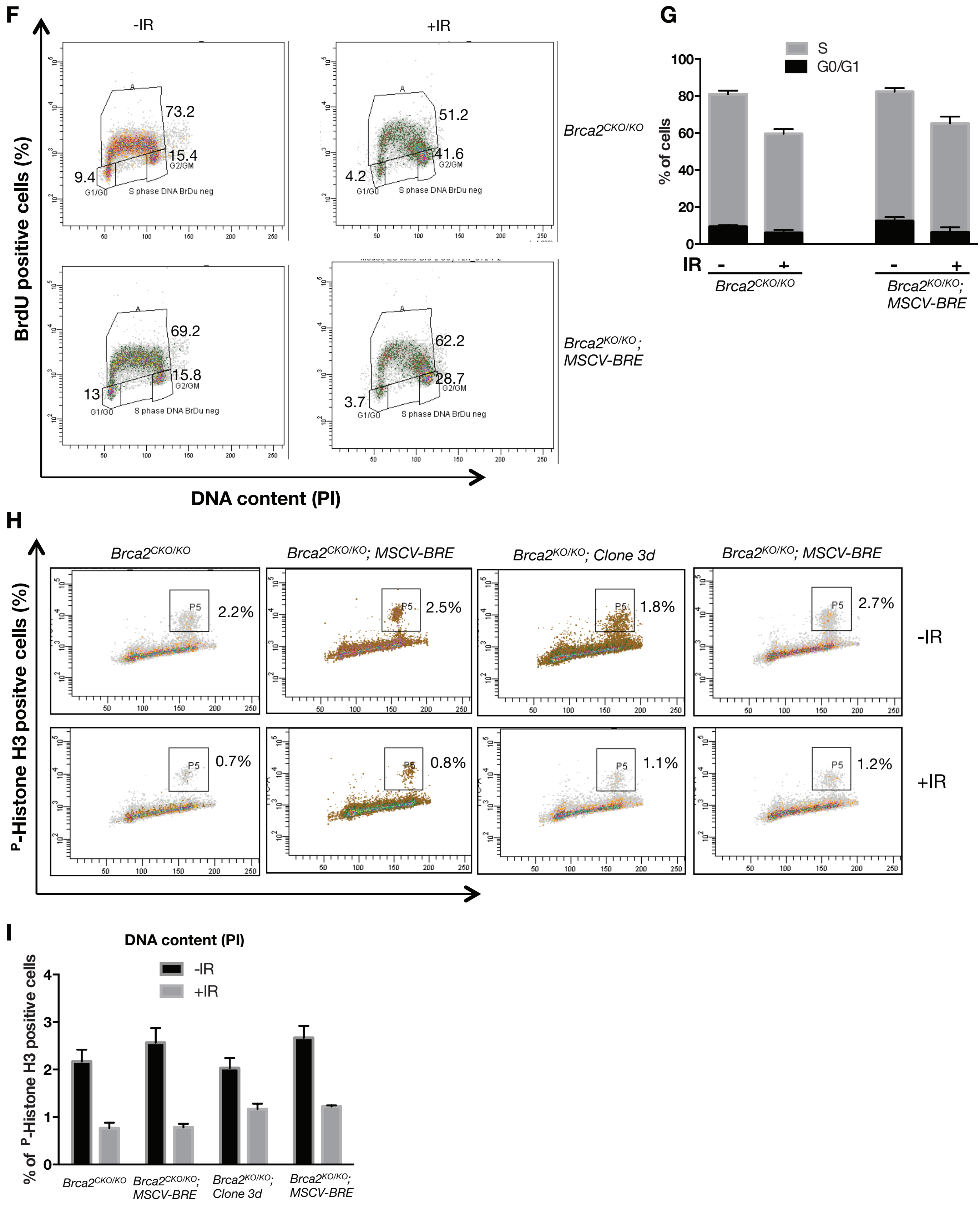
F
-IR
+IR
BrdU positive cells (%)
73.2
15.4
9.4
51.2
41.6
4.2
69.2
15.8
13
62.2
28.7
3.7
G1/G0
S phase DNA BrDu neg
G2/GM
Brca2CKO/KO
Brca2KO/KO; MSCV-BRE
DNA content (PI)
G
S
G0/G1
% of cells
IR
Brca2CKO/KO
Brca2KO/KO; MSCV-BRE
H
Brca2CKO/KO
Brca2CKO/KO; MSCV-BRE
Brca2KO/KO; Clone 3d
Brca2KO/KO; MSCV-BRE
P5
2.2%
2.5%
1.8%
2.7%
0.7%
0.8%
1.1%
1.2%
-IR
+IR
P-Histone H3 positve cells (%)
I
DNA content (PI)
-IR
+IR
% of P-Histone H3 positive cells
Brca2CKO/KO
Brca2CKO/KO; MSCV-BRE
Brca2KO/KO; Clone 3d
Brca2KO/KO; MSCV-BRE

Supplementary Figure 2. BRCA2 functions are unaffected by BRE overexpression. (**A**) Immunoblot showing the expression of RAD51 in mES cells. Control: GAPDH blot. (**B-D**) Drug sensitivity assay of mES cells using XTT assay for cell survival was plotted after treating the cells with different doses of olaparib (**B**) cisplatin (**C**) or ionizing radiation (**D**). All values are represented as mean ± s.d. Tables on the right show the *P*-values compared to control cells using two-tailed paired t-test and tabulated in Supplentary table 2. (**E**) Western blot demonstrating TRP53 level in mES cells at different time after IR. GAPDH was used as control. (**F**) Analysis of BrdU incorporation in asynchronously growing mES cells after γ-radiation using Flow cytometry. DNA content was measured by propidium iodide (PI) staining. (**G**) Plot showing the percentage of cells incorporated BrdU. Mean values of three independent experiments were plotted. Error bars represent s.d. values. (**H**) Flow cytometric analysis of asynchronously growing mES cells stained for phosphorylated histone H3 after irradiation. PI was used to stain DNA. (**I**) Graph showing the percentage of mitotic cells (positive for phospho-histone H3). Values represent mean ± s.d.

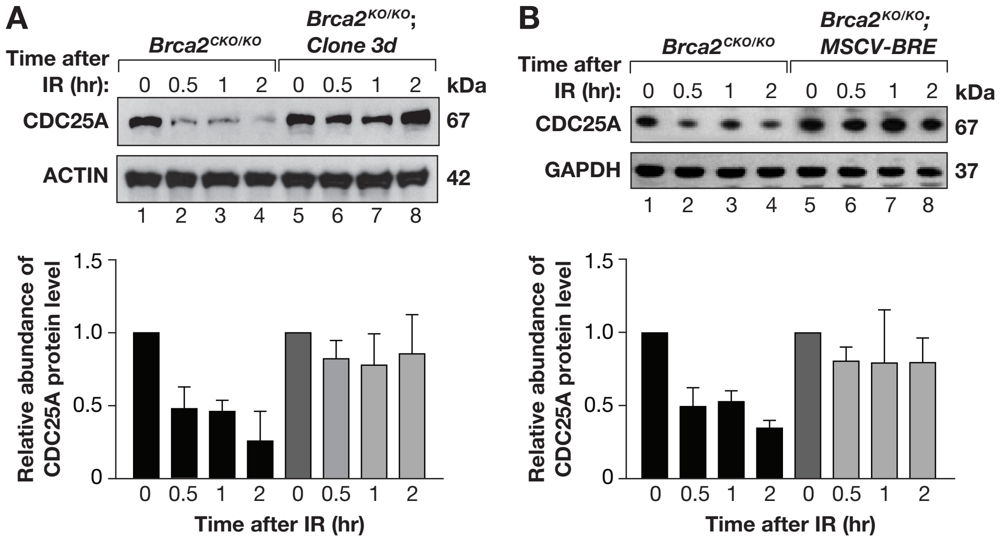

Supplementary Figure 3. IR induced CDC25A degradation in BRE overexpressed BRCA2 deficient mES cells. Western blot showing the abundance of CDC25A at different times after exposing the cells to 6 Gy IR in *Brca2*$^{KO/KO}$;Clone 3d (A) and $^{\mathrm{Brca2KO/KO;MSCV\text{-}BRE}}$ (B) mES cells. *Brca2*$^{CKO/KO}$ cells was used as control. GAPDH was used as a loading control. Numbers below indicate lane numbers. Histogram shows quantification of CDC25A band intensity for each cell lines. Average of relative band intensities from three independent experiments were represented. Error bars represent s.d. P values were calculated using paired two-tailed t-test tabulated in Supplementary Table 2.

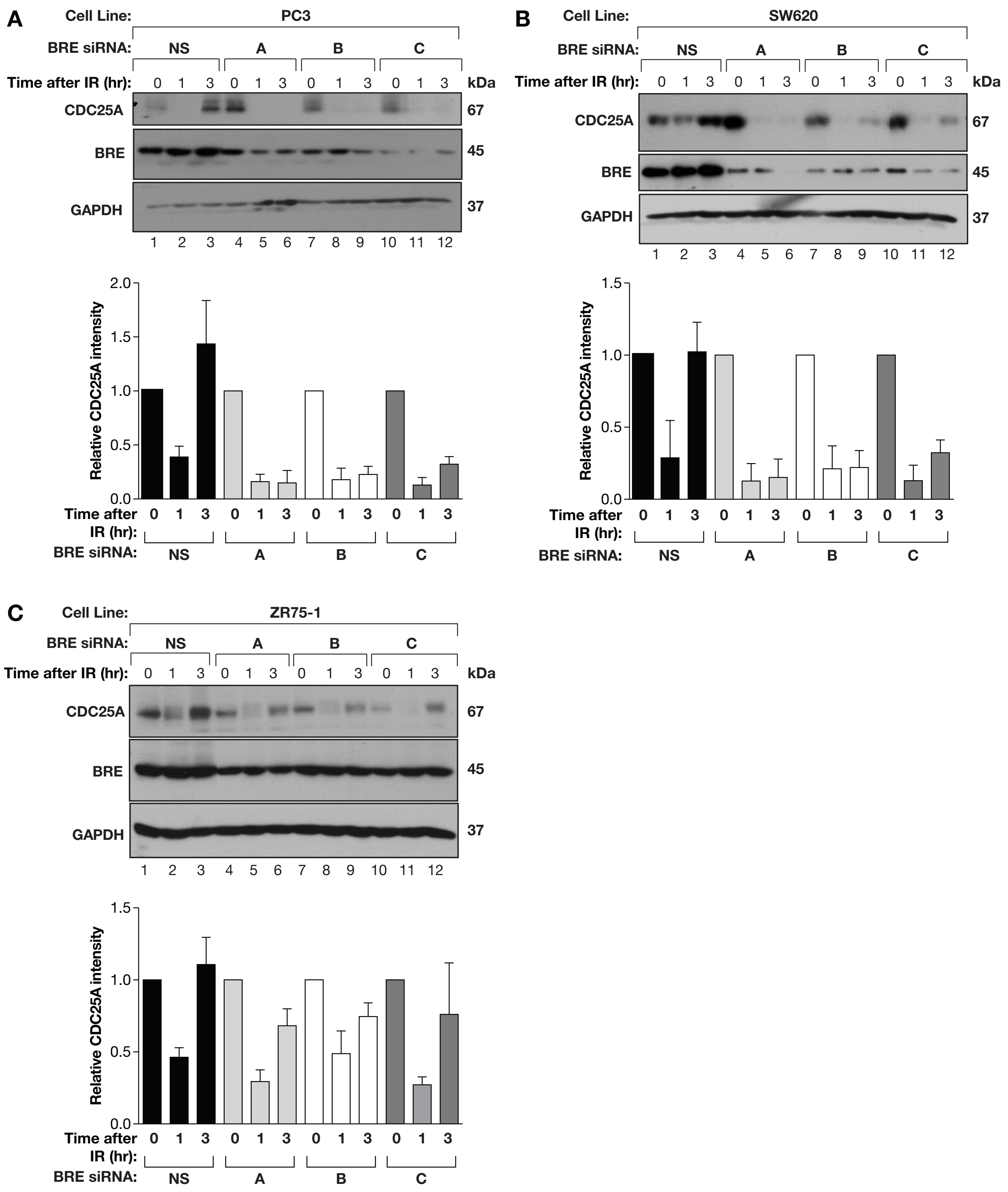
A
Cell Line:
PC3
BRE siRNA:
NS
A
B
C
Time after IR (hr):
0 1 3 0 1 3 0 1 3 0 1 3
kDa
CDC25A
67
BRE
45
GAPDH
37
1 2 3 4 5 6 7 8 9 10 11 12
Relative CDC25A intensity
2.0
1.5
1.0
0.5
0.0
Time after IR (hr):
0 1 3 0 1 3 0 1 3 0 1 3
BRE siRNA:
NS
A
B
C
B
Cell Line:
SW620
BRE siRNA:
NS
A
B
C
Time after IR (hr):
0 1 3 0 1 3 0 1 3 0 1 3
kDa
CDC25A
67
BRE
45
GAPDH
37
1 2 3 4 5 6 7 8 9 10 11 12
Relative CDC25A intensity
1.5
1.0
0.5
0.0
Time after IR (hr):
0 1 3 0 1 3 0 1 3 0 1 3
BRE siRNA:
NS
A
B
C
C
Cell Line:
ZR75-1
BRE siRNA:
NS
A
B
C
Time after IR (hr):
0 1 3 0 1 3 0 1 3 0 1 3
kDa
CDC25A
67
BRE
45
GAPDH
37
1 2 3 4 5 6 7 8 9 10 11 12
Relative CDC25A intensity
1.5
1.0
0.5
0.0
Time after IR (hr):
0 1 3 0 1 3 0 1 3 0 1 3
BRE siRNA:
NS
A
B
C

Supplementary Figure 4. IR induced CDC25A degradation in different tumor cell lines after BRE knockdown. Immunoblot after transfection of non-specific (NS) or three different BRE siRNAs (A, B or C) into breast cancer cell line PC3 (**A**), colorectal cancer cell line SW620 (**B**) and prostate cancer cell line ZR75-1 (**C**). GAPDH used as control. Lower panel on each section shows the average relative CDC25A band intensity at corresponding time points for three independent experiments. Error bars are represented by s.d values. Relative intensity was measured for each set of siRNA treatment by dividing GAPDH normalized CDC25A intensity at particular time point with GAPDH normalized CDC25A intensity at 0 hr time point of the particular set. Paired two tailed t-test was used to measure *P* values and are tabulated in Supplementary table 2.

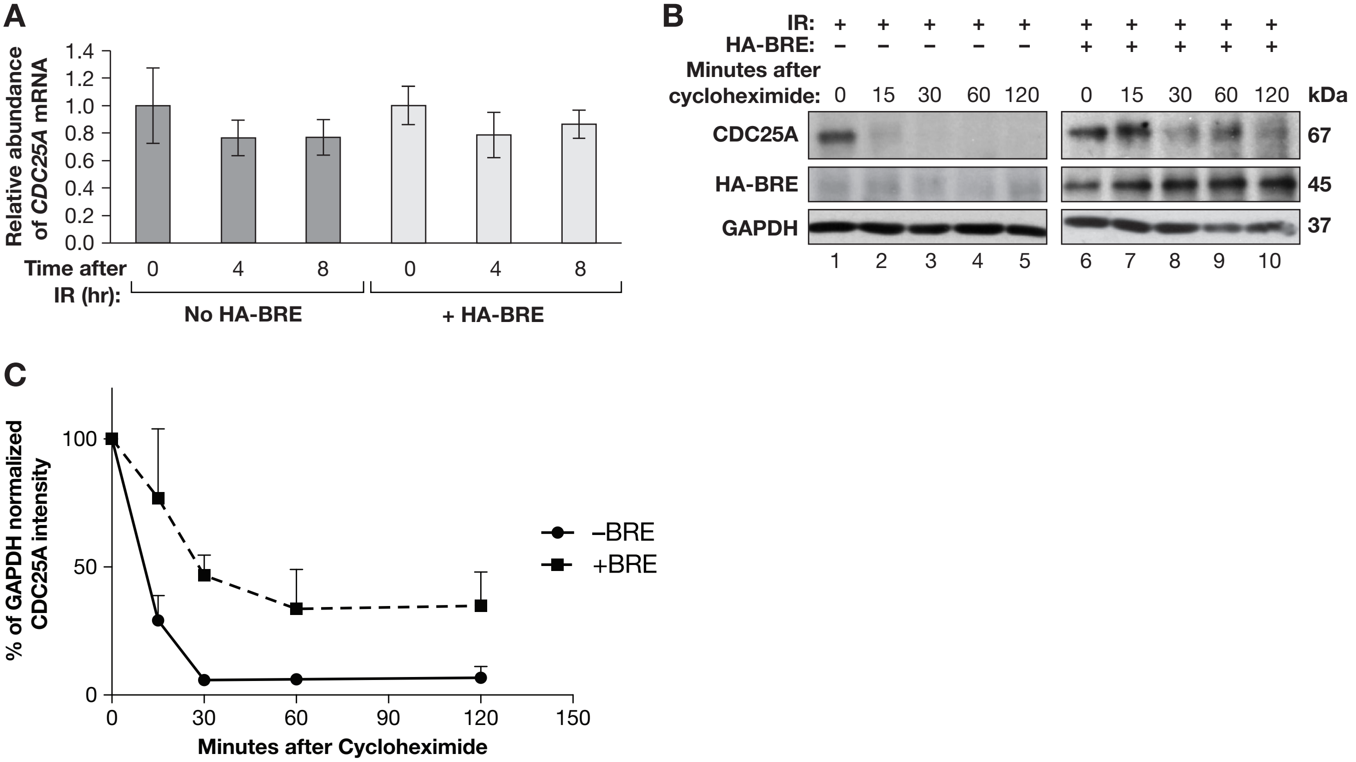

Supplementary Figure 5. BRE overexpression regulates CDC25A post-transnationally. (A) Quantification of CDC25A mRNA levels in MCF7 cells by real time RT-PCR at 0, 4 and 8 hrs after IR in presence and absence of exogenous HA-BRE expression. (B) Representative western blot analysis showing CDC25A levels in MCF7 cells at different time points after 50 µg/ml cycloheximide treatment without (left panel) and with (right panel) exogenous HA-BRE expression. Cells were treated with 6 Gy IR. (C) Histogram representing the quantification of CDC25A abundance relative to GAPDH. Aberage of three independent experiments were plotted and error bars represents s.d. values. P values measured by paired two tailed t-tests for points are shown in Supplementary Table 2.

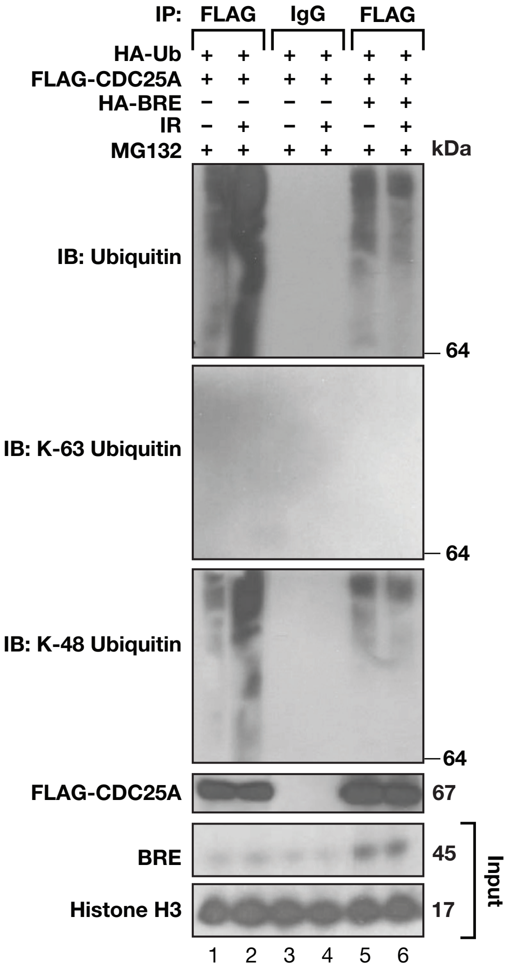

Supplementary Figure 6. BRE overexpression affects IR induced K-48 linked ubiquitylation of CDC25A. Top blot represents total polyubiquitylation of FLAG-CDC25A in cells harvested 4hr after 6 Gy IR in absence (lanes 1-4) or presence (lanes 5, 6) of exogenous BRE. CDC25A was immunoprecipitated using FLAG antibody (lanes 1,2, 5, 6) and IgG was used as control (lanes 3, 4). Results of immunoblotting with K-63 ubiquitin and K-48 ubiquitin antibodies are represented in second and third panels. The same blot was stripped and re-incubated with specific antibodies sequentially. Input controls are shown at the bottom.

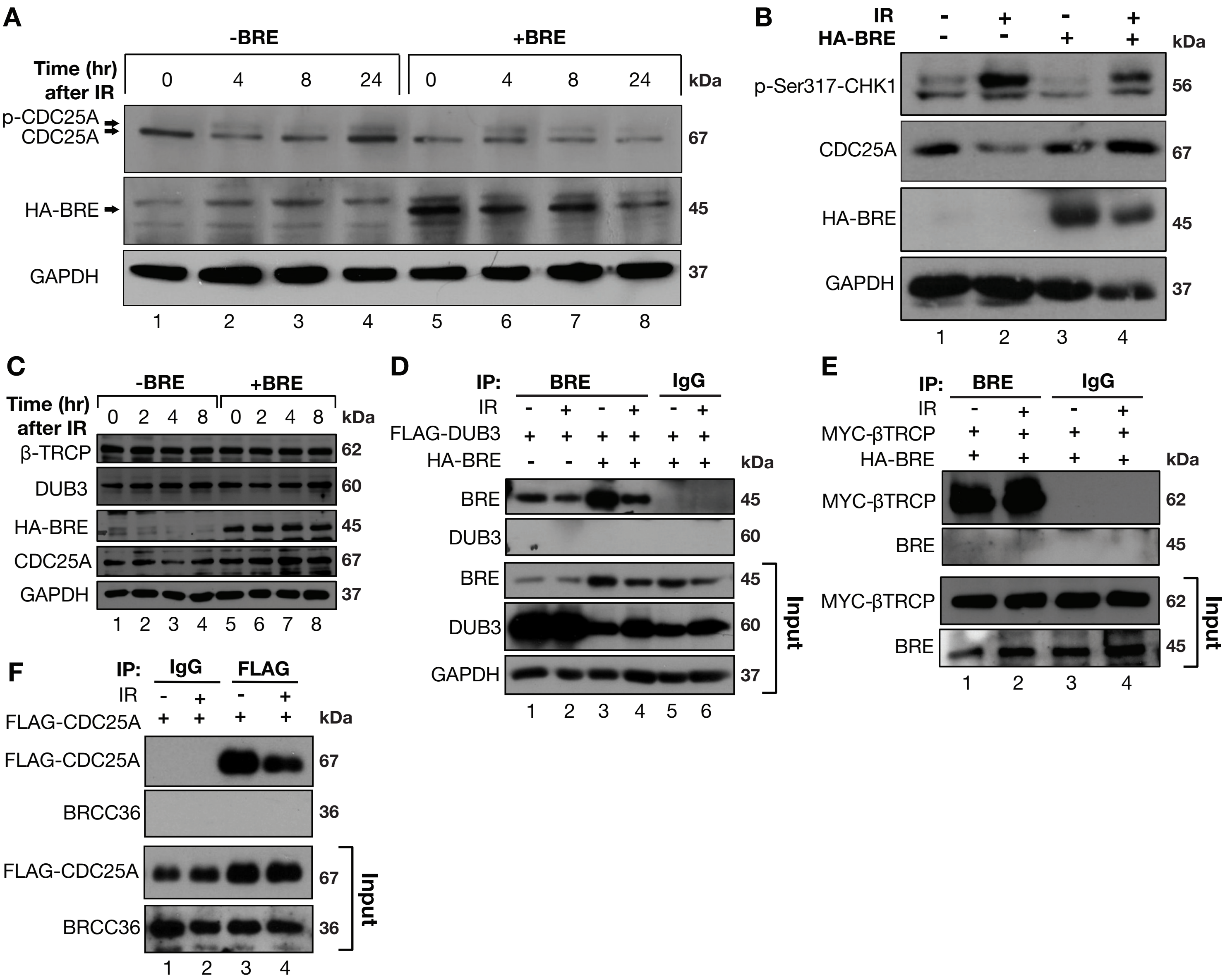

Supplementary Figure 7. BRE overexpression does not affect known regulators of CDC25A. (A) Western blot showing phosphorylated CDC25A in MCF7 cells at different times after IR. (B) Western blot showing CHK1 phosphorylation at Ser 317 in irradiated (4 hrs after 6 Gy radiation) and non-irradiated MCF7 cells. (C) Western blot showing the abundance of β-TRCP and DUB3 in MCF7 cells at different times after 6 Gy γ-radiation. (D) Co-immunoprecipitation of DUB3 and BRE in MCF7 cells. Anti-BRE antibody was used to pull down. (E) Co-immunoprecipitation of Myc tagged-β-TRCP and BRE in MCF7 cells. (F) Co-immunoprecipitation of BRCC36 and FLAG-CDC25A in MCF7 cells. Cells were treated after irradiation with 5 μM MG132 to prevent protein degradation.

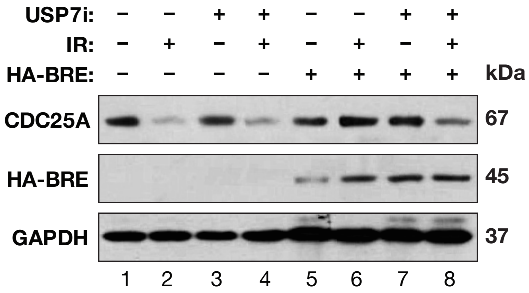

Supplementary Figure 8. IR induced CDC25A degradation in presence of USP7 chemical inhibitor. Immunoblot representing IR induced CDC25A degradation in presence (lanes 5-8) and absence of exogenous HA-BRE (lanes 1-4) in combination with either DMSO control (lanes 1, 2, 5, 6) or 10 μM P5091 [USP7 inhibitor] (lanes 3, 4, 7, 8). Cells were subjected to 6Gy IR and harvested at 0 hr (-) or 4 hr (+) after IR. GAPDH was used as loading control.

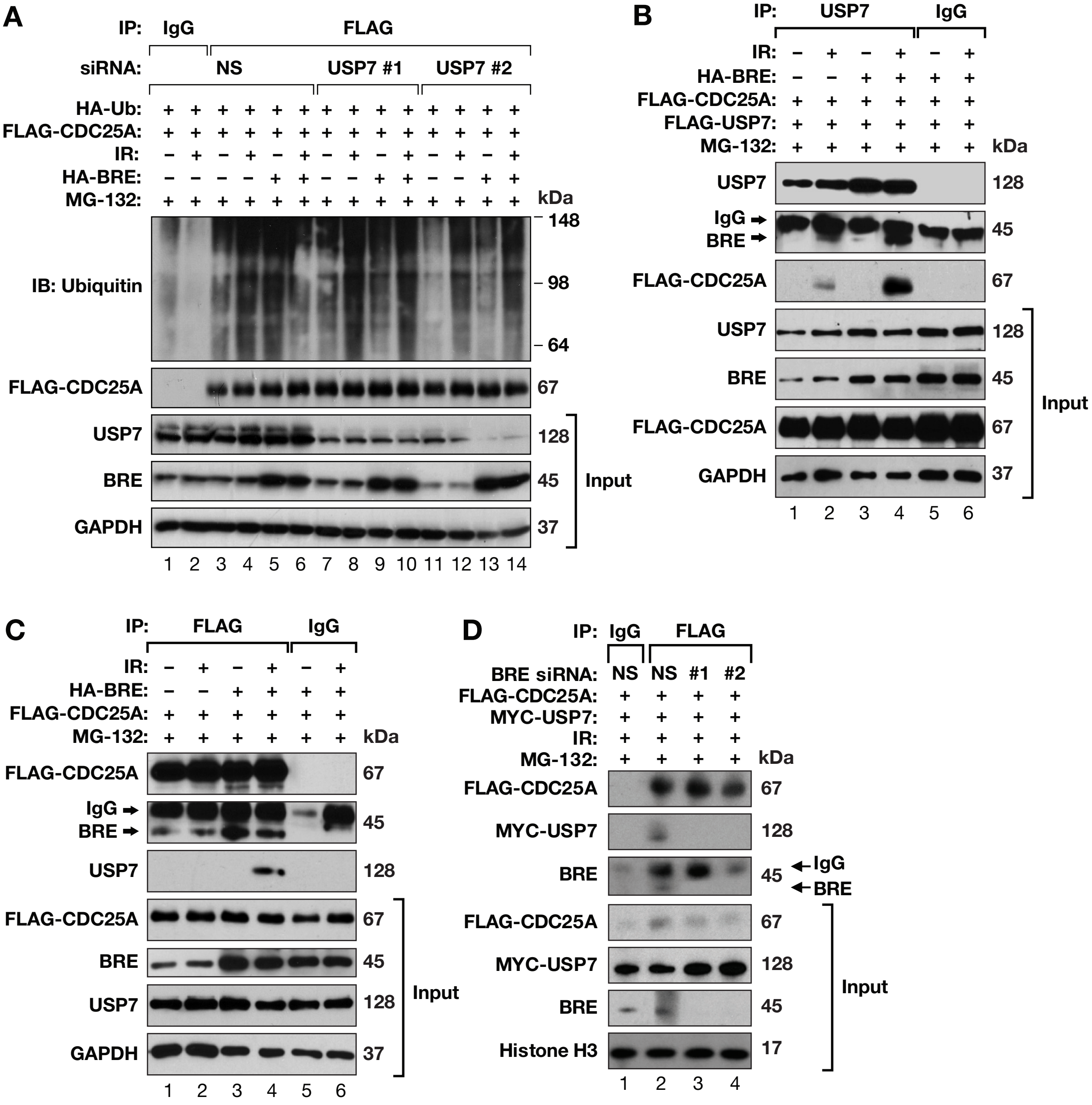
A
IP: IgG FLAG
siRNA: NS USP7 #1 USP7 #2
HA-Ub:
FLAG-CDC25A:
IR:
HA-BRE:
MG-132:
kDa
IB: Ubiquitin
148
98
64
FLAG-CDC25A 67
USP7 128
BRE 45
GAPDH 37
Input
1 2 3 4 5 6 7 8 9 10 11 12 13 14
B
IP: USP7 IgG
IR:
HA-BRE:
FLAG-CDC25A:
FLAG-USP7:
MG-132:
kDa
USP7 128
IgG
BRE
45
FLAG-CDC25A 67
USP7 128
BRE 45
FLAG-CDC25A 67
GAPDH 37
Input
1 2 3 4 5 6
C
IP: FLAG IgG
IR:
HA-BRE:
FLAG-CDC25A:
MG-132:
kDa
FLAG-CDC25A 67
IgG
BRE
45
USP7 128
FLAG-CDC25A 67
BRE 45
USP7 128
GAPDH 37
Input
1 2 3 4 5 6
D
IP: IgG FLAG
BRE siRNA: NS NS #1 #2
FLAG-CDC25A:
MYC-USP7:
IR:
MG-132:
kDa
FLAG-CDC25A 67
MYC-USP7 128
BRE 45
IgG
BRE
FLAG-CDC25A 67
MYC-USP7 128
BRE 45
Histone H3 17
Input
1 2 3 4

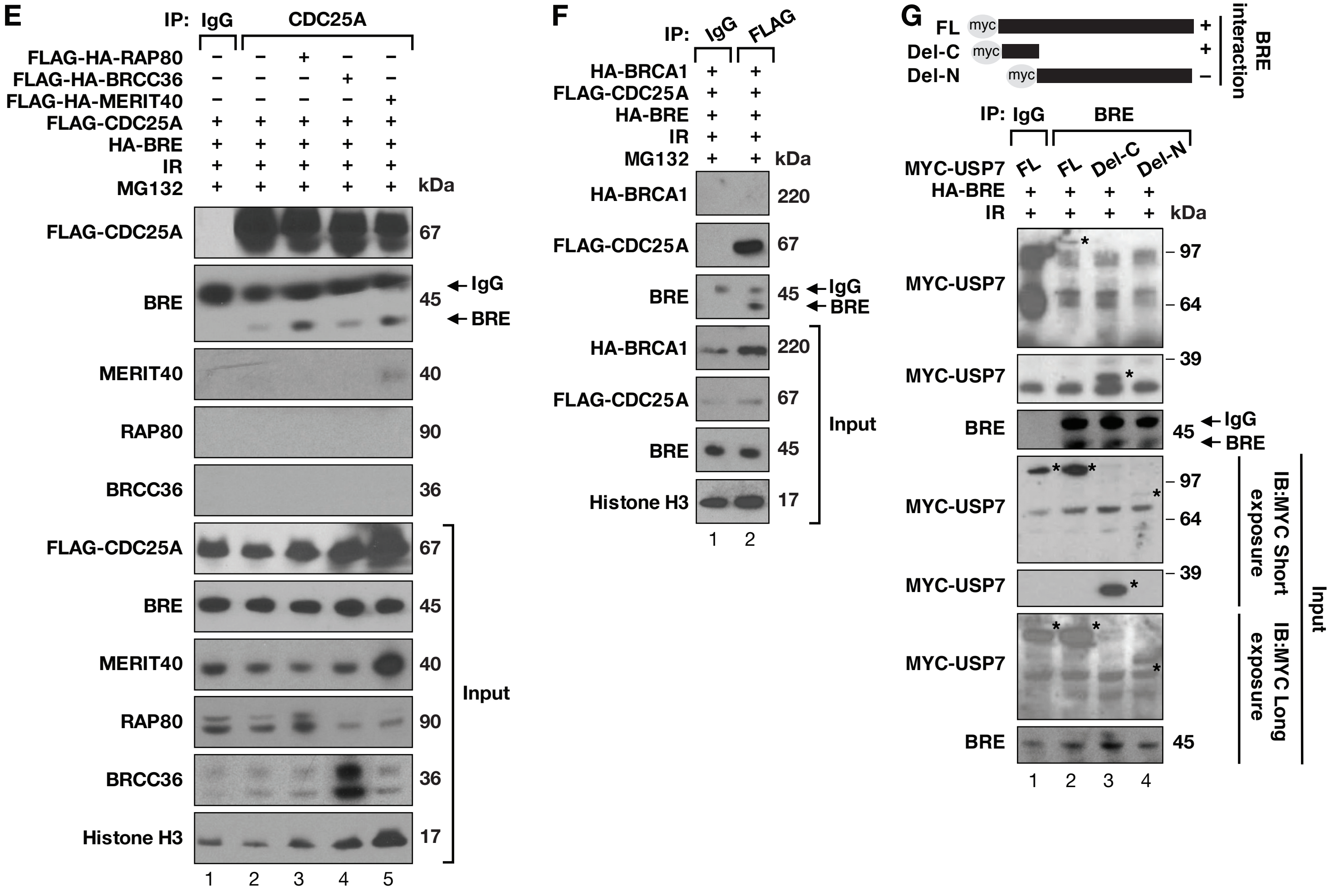

Supplementary Figure 9. Polyubiquitylation and interaction of CDC25A with BRE and USP.

(A) Ubiquitylation of FLAG-CDC25A in MCF7 cells after transfection of either non-specific (NS, lanes 1-6) or USP7 specific (USP7#1, lanes 7-10 and USP7#2, lanes 11-14) siRNAs in presence (lanes 5, 6, 9, 10, 13, 14) and absence of exogenous HA-BRE. Cells were harvested at 0 hr (-) or 4 hr (+) after 6 Gy IR. IP was performed using anti-FLAG antibody (lanes 3-14) and IgG antibody (lanes 1, 2) as control. (B) Immunoblot of co-IP from FLAG-USP7, FLAG-CDC25A and HA-BRE expressing MCF7 cells using anti-USP antibody (lanes 1-4) and IgG antibody (lanes 5-6). Cells are treated with MG132 (5 μM) after irradiation for 2 hr. (C) Co-immunoprecipitation of FLAG-CDC25A, BRE and endogenous USP7. Anti-FLAG antibody was used to pull down CDC25A

(lanes 1-4) and IgG antibody (lanes 5-6) was used as control. Cells were cultured in presence of MG132 (5μM) after irradiation for 2 hr. (**D**) Immunoblot of co-IP from MYC-USP7, FLAG-CDC25A expressing MCF7 cells in presence of either non-specific
(NS) or two independent BRE specific siRNAs (BRE #1 and BRE #2) using anti-FLAG antibody (lanes 2-4) and IgG antibody (lane 1). Cells were treated with MG132 (5 μM) for 2 hours after irradiation for (**E**) Western blot of co-immunoprecipitation from cells expressing FLAG-CDC25A and HA-BRE and either FLAG-HA-RAP80 or FLAG-HA-BRCC36 or FLAG-HA-MERIT40 using anti-CDC25A antibody (lanes 2-5) or IgG antibody (lane 1). Cells were treated with 5 μM MG132 for 2 hr after irradiation. (**F**) Immunoblot of co-IP from cells expressing FLAG-CDC25A, HA-BRE and HA-BRCA1 using anti-FLAG antibody. Cells were subjected to 5 μM MG132 treatment after IR for 2 hr. (**G**) Co-immunopricipitation of BRE and different deletion mutants of USP7 tagged with MYC-epitope at N-terminus from irradiated HEK293 cells using BRE antibody. Top panel shows the schematic presentation of different deletion mutants of USP7. Stars indicate different MYC-USP7 proteins.

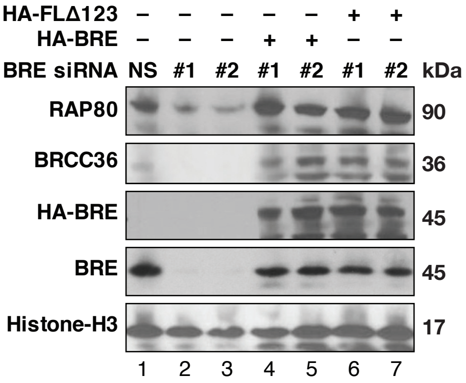

Supplementary Figure 10. Effect of HA-BRE mutant defective in USP7 binding (FLΔ123) on stability of BRISC complex proteins after knockdown of endogenous BRE. Immunoblot showing the level of RAP80 and BRCC36 proteins in NIH3T3 cells either in presence of HA-BRE (full length human BRE cDNA) or HA- FLΔ123 (mutant human BRE cDNA) after knockdown of endogenous BRE. Two independent siRNAs (Bre #1 and Bre #2) were used. NS represents non-specific siRNA. Histone H3 was used as loading control.

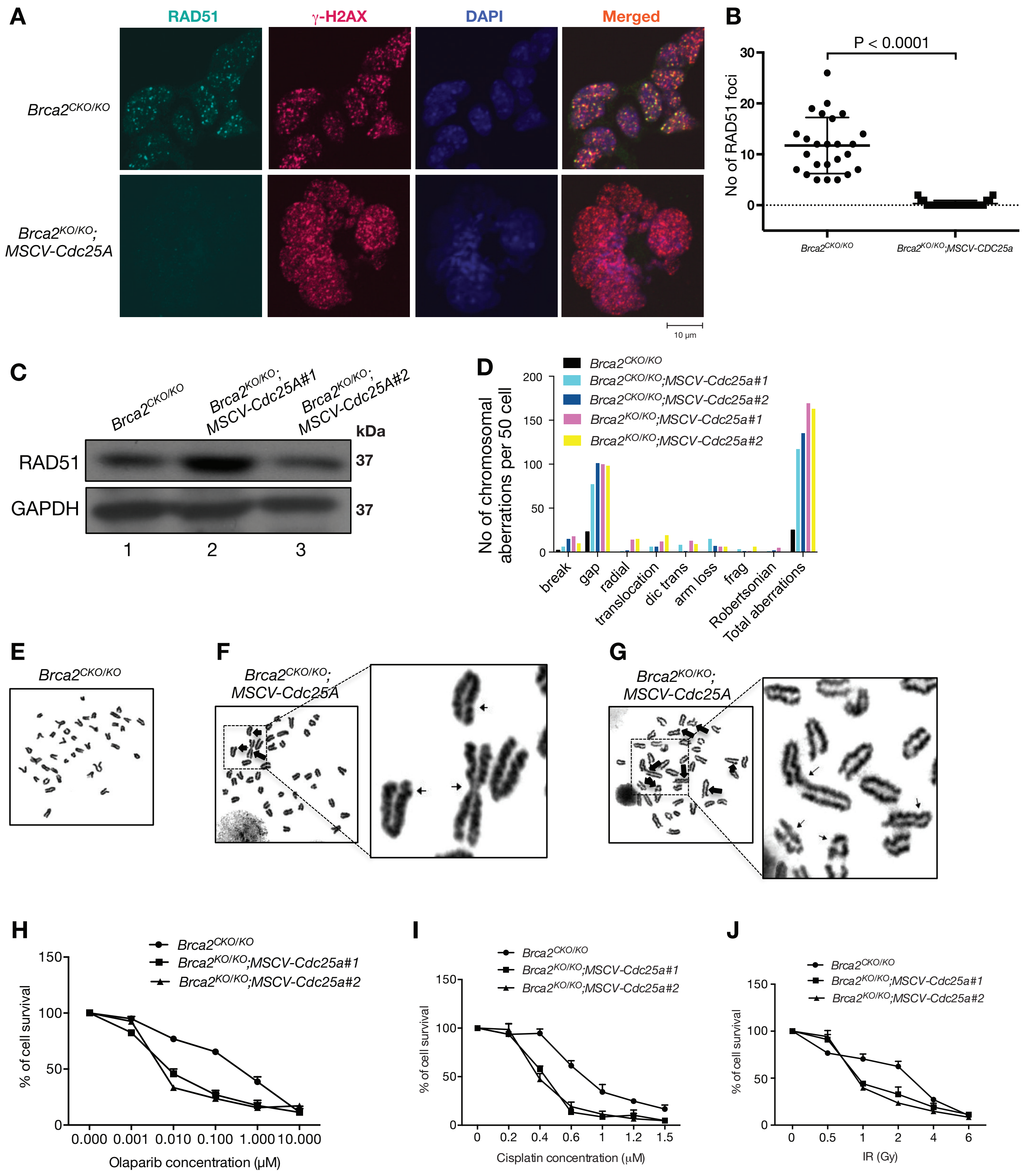
A
RAD51
γ-H2AX
DAPI
Merged
Brca2CKO/KO
Brca2KO/KO; MSCV-Cdc25A
10 µm
B
P < 0.0001
No of RAD51 foci
Brca2CKO/KO
Brca2KO/KO;MSCV-CDC25a
C
Brca2CKO/KO
Brca2KO/KO; MSCV-Cdc25A#1
Brca2KO/KO; MSCV-Cdc25A#2
kDa
RAD51
GAPDH
37
37
1
2
3
D
Brca2CKO/KO
Brca2CKO/KO;MSCV-Cdc25a#1
Brca2CKO/KO;MSCV-Cdc25a#2
Brca2KO/KO;MSCV-Cdc25a#1
Brca2KO/KO;MSCV-Cdc25a#2
No of chromosomal aberrations per 50 cell
break
gap
radial
translocation
dic trans
arm loss
frag
Robertsonian
Total aberrations
E
Brca2CKO/KO
F
Brca2CKO/KO; MSCV-Cdc25A
G
Brca2KO/KO; MSCV-Cdc25A
H
Brca2CKO/KO
Brca2KO/KO;MSCV-Cdc25a#1
Brca2KO/KO;MSCV-Cdc25a#2
% of cell survival
Olaparib concentration (µM)
I
Brca2CKO/KO
Brca2KO/KO;MSCV-Cdc25a#1
Brca2KO/KO;MSCV-Cdc25a#2
% of cell survival
Cisplatin concentration (µM)
J
Brca2CKO/KO
Brca2KO/KO;MSCV-Cdc25a#1
Brca2KO/KO;MSCV-Cdc25a#2
% of cell survival
IR (Gy)

Supplementary Figure 11. DNA repair assays for *Brca2$^{KO/KO}$* ES cells rescued by CDC25A overexpression. (**A**) RAD51 foci formation in response to DNA damage. Green: RAD51, Red: γ-H2AX, Blue: nuclei are stained with DAPI. Yellow foci in merged image marks that RAD51 foci co-localize with γ-H2AX foci. (**B**) Histogram showing the quantification of RAD51 foci after ionizing radiation. For each genotype, 25 nuclei were counted. Error bars represent the mean ± s.d. (**C**) Western blot detecting RAD51 protein. GAPDH was used as control. (**D**) Graph representing number of different chromosomal aberrations as well as total aberrations. (**E-G**) Karyotype analysis of ES cells. (**E**) *Brca2$^{CKO/KO}$* (**F**) *Brca2$^{CKO/KO}$; MSCV-Cdc25A* (**G**) *Brca2$^{KO/KO}$; MSCV-Cdc25A* cells representative metaphase spread. Arrows indicate different aberrations. (**H-J**) Sensitivity to different DNA damaging agents. Plots showing (**H**) Olaparib (**I**) Cisplatin (**J**) IR treated cell survival as measured by XTT assay. All values shown are mean of three independent experimental values. Error bars represent s.d. *P*-values (two-tailed, paired) are shown for each point in the Supplementary Table 2.

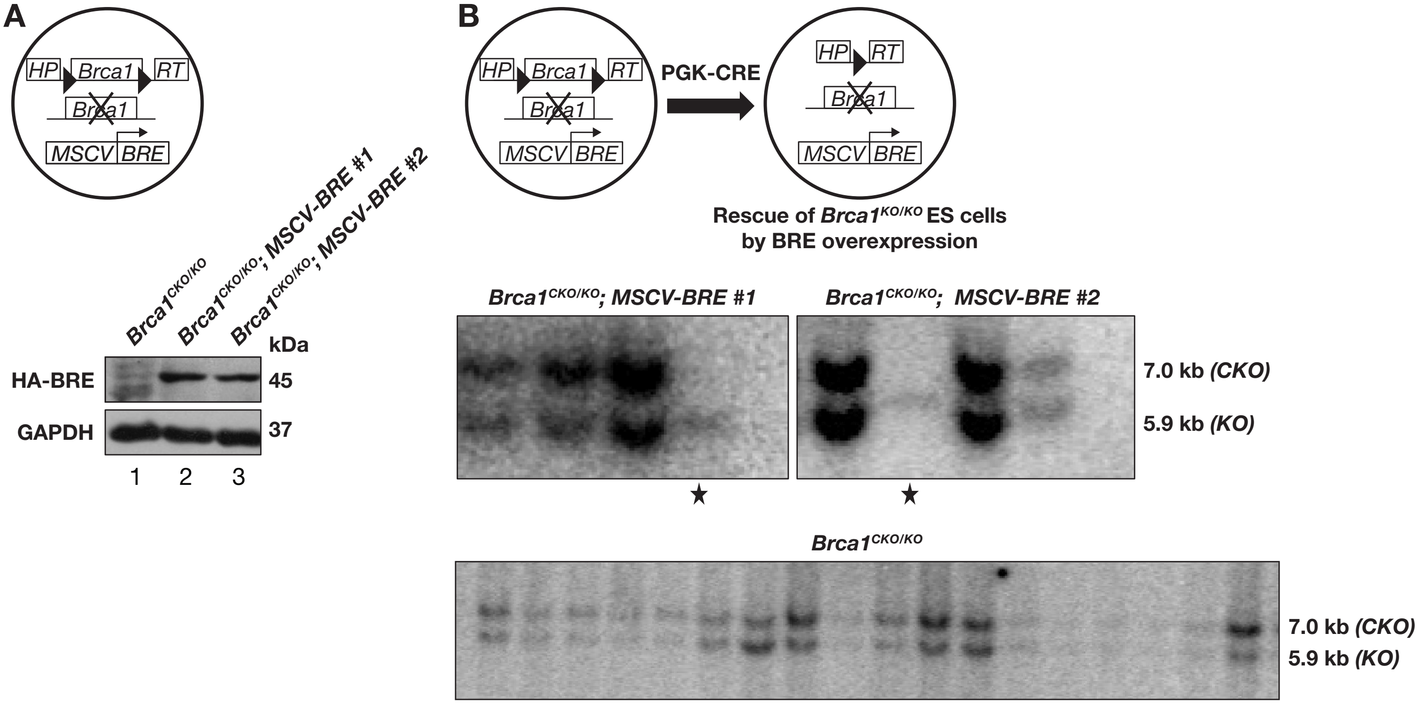

Supplementary Figure 12. BRE overexpression supports survival of *Brca1*$^{KO/KO}$ ES cells. (**A**) Western blot showing the expression of HA-tagged BRE in *Brca1* mutant mouse ES cells. Scheme for expressing *HA-BRE* is shown at top. (**B**) Southern blot analysis of HAT resistant ES cell colonies after CRE-mediated deletion of conditional allele in *Brca1*$^{CKO/KO}$*; MSCV-BRE* ES cells to identify *Brca1*$^{KO/KO}$ clones (marked with solid stars), upper band: conditional allele (*CKO*); lower band: knock-out allele (*KO*). *Brca1*$^{CKO/KO}$ cells were used as control.

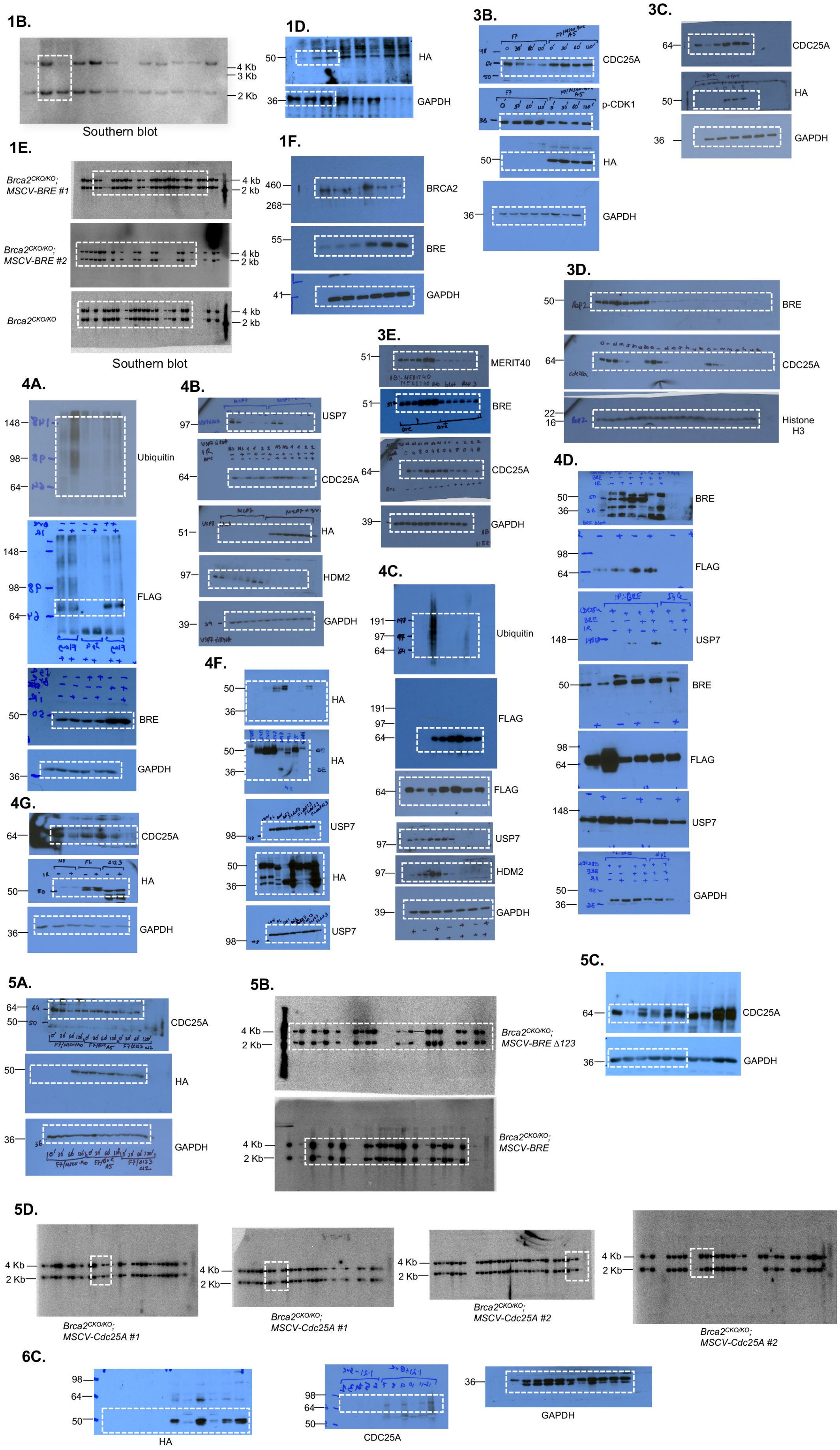
1B.
4 Kb
3 Kb
2 Kb
Southern blot
1D.
HA
GAPDH
3B.
CDC25A
p-CDK1
HA
GAPDH
3C.
CDC25A
HA
GAPDH
1E.
Brca2CKO/KO; MSCV-BRE #1
Brca2CKO/KO; MSCV-BRE #2
Brca2CKO/KO
4 kb
2 kb
Southern blot
1F.
BRCA2
BRE
GAPDH
3D.
BRE
CDC25A
Histone H3
3E.
MERIT40
BRE
CDC25A
GAPDH
4A.
Ubiquitin
FLAG
BRE
GAPDH
4B.
USP7
CDC25A
HA
HDM2
GAPDH
4D.
BRE
FLAG
USP7
BRE
FLAG
USP7
GAPDH
4C.
Ubiquitin
FLAG
FLAG
USP7
HDM2
GAPDH
4F.
HA
HA
USP7
HA
USP7
4G.
CDC25A
HA
GAPDH
5A.
CDC25A
HA
GAPDH
5B.
4 Kb
2 Kb
Brca2CKO/KO; MSCV-BRE Δ123
Brca2CKO/KO; MSCV-BRE
5C.
CDC25A
GAPDH
5D.
Brca2CKO/KO; MSCV-Cdc25A #1
Brca2CKO/KO; MSCV-Cdc25A #1
Brca2CKO/KO; MSCV-Cdc25A #2
Brca2CKO/KO; MSCV-Cdc25A #2
6C.
HA
CDC25A
GAPDH

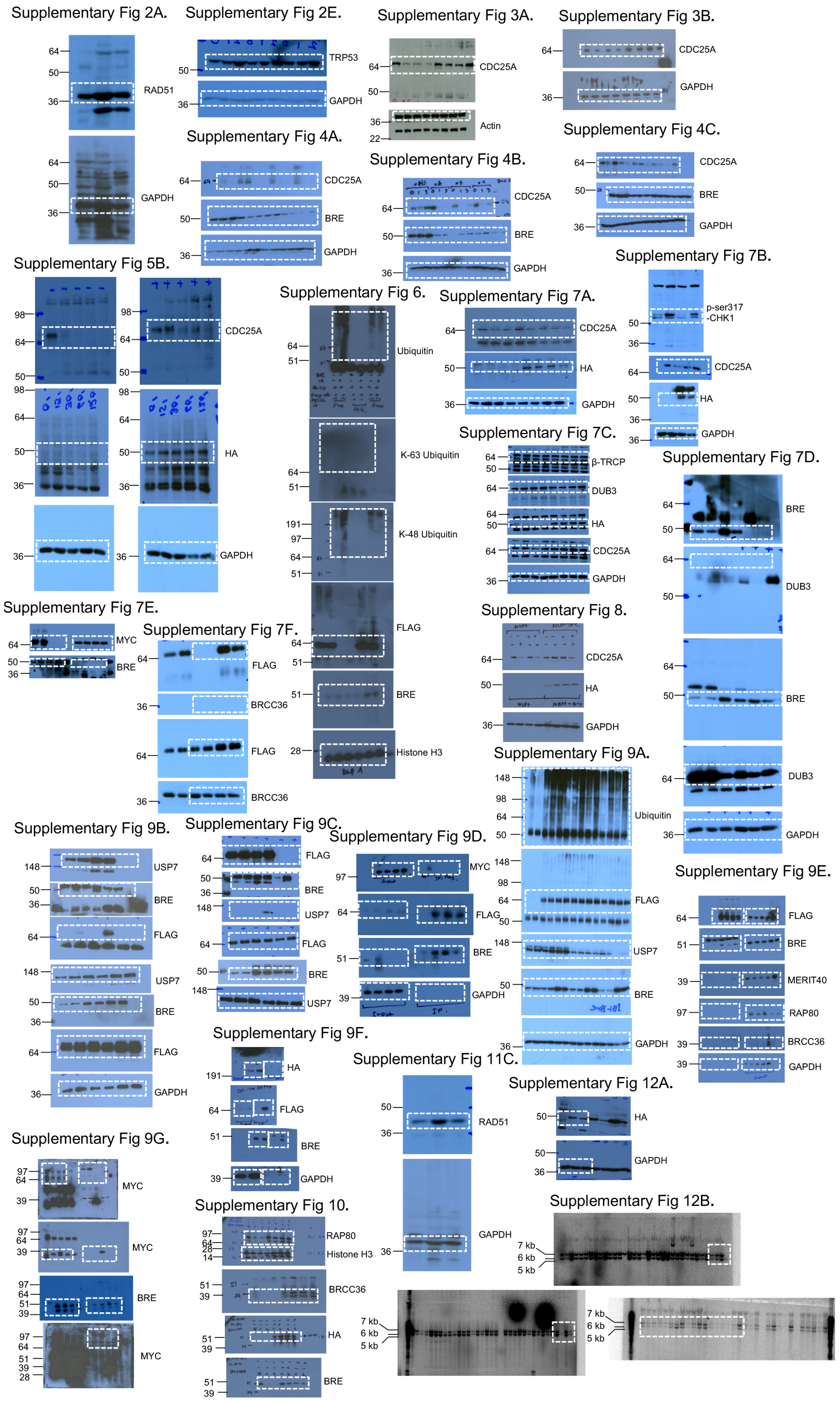

Supplementary Fig 2A.
RAD51
GAPDH
Supplementary Fig 2E.
TRP53
GAPDH
Supplementary Fig 3A.
CDC25A
Actin
Supplementary Fig 3B.
CDC25A
GAPDH
Supplementary Fig 4A.
CDC25A
BRE
GAPDH
Supplementary Fig 4B.
CDC25A
BRE
GAPDH
Supplementary Fig 4C.
CDC25A
BRE
GAPDH
Supplementary Fig 5B.
CDC25A
HA
GAPDH
Supplementary Fig 6.
Ubiquitin
K-63 Ubiquitin
K-48 Ubiquitin
FLAG
BRE
Histone H3
Supplementary Fig 7A.
CDC25A
HA
GAPDH
Supplementary Fig 7B.
p-ser317
-CHK1
CDC25A
HA
GAPDH
Supplementary Fig 7C.
β-TRCP
DUB3
HA
CDC25A
GAPDH
Supplementary Fig 7D.
BRE
DUB3
BRE
DUB3
GAPDH
Supplementary Fig 7E.
MYC
BRE
Supplementary Fig 7F.
FLAG
BRCC36
FLAG
BRCC36
Supplementary Fig 8.
CDC25A
HA
GAPDH
Supplementary Fig 9A.
Ubiquitin
FLAG
USP7
BRE
GAPDH
Supplementary Fig 9B.
USP7
BRE
FLAG
USP7
BRE
FLAG
GAPDH
Supplementary Fig 9C.
FLAG
BRE
USP7
FLAG
BRE
USP7
Supplementary Fig 9D.
MYC
FLAG
BRE
GAPDH
Supplementary Fig 9E.
FLAG
BRE
MERIT40
RAP80
BRCC36
GAPDH
Supplementary Fig 9F.
HA
FLAG
BRE
GAPDH
Supplementary Fig 9G.
MYC
MYC
BRE
MYC
Supplementary Fig 10.
RAP80
Histone H3
BRCC36
HA
BRE
Supplementary Fig 11C.
RAD51
GAPDH
Supplementary Fig 12A.
HA
GAPDH
Supplementary Fig 12B.
7 kb
6 kb
5 kb

Supplementary Figure 13. Uncropped blots of the figures presented. White dashed boxes mark the region shown in figures. Position of markers are included left and the antibodies used are marked on right.

Supplementary Table 1.

Sample	CDC25A mRNA	BRCA2 mutation	BRCA1 mutation
MB-0062	1.0559	K68T	
MB-0163	1.5728		S1551C
MB-0420	1.1148		V1654Cfs*4
MB-0664	0.531	C3069Lfs*5	
MB-0234	-0.0932		E914K
MB-0532	0.8357		Y1127H
MB-4303	0.6386	H2455R	
MB-2617	0.3899		Y1127H
MB-0320	-1.2436		D420G
MB-2835	-0.8339	D3378V	
MB-0346	1.4995		R1737T
MB-2847	1.5074	T3310Nfs*17	
MB-0360	-0.5614	D1911N	
MB-2922	0.3509		I216S
MB-3026	0.1392	A737V	
MB-3057	0.3022	V741L	
MB-3228	-0.207	R2973C	
MB-4938	0.139	L2654Ffs*3	
MB-0569	0.4499	S2133*	
MB-5062	1.3542		L1439F
MB-5070	1.6548		T150Pfs*13
MB-5098	-0.7176	V2166L	
MB-5088	-0.1476		I946V
MB-5107	0.3718		E1494Kfs*11
MB-5130	-1.0935	V3365_S3366insELY,S3366Mfs*2	
MB-5166	1.3267		R613K
MB-4426	-0.0932	K280*	
MB-2735	0.5526		W372R
MB-4627	-0.4048		S153R
MB-2834	0.6128	E1120V	
MB-4785	0.6948	S231R	
MB-4791	0.476	D3378N	
MB-5530	0.0924	Y3098*	
MB-5593	0.4862	V2687I	

MB-5602	0.5768		E1836K
MB-5646	-0.6117	Q3066*	
MB-6007	1.0559		E391K
MB-5152	0.3499		M1827L
MB-5164	-0.5768		E143K
MB-6060	1.027	A3205P,A3205V	S282*
MB-6062	2.2625		I171M
MB-5184	-1.074	V3365_S3366insELY,S3366Mfs*2	
MB-5196	1.1771	E1581Q	
MB-5213	0.6428	K1875N	
MB-4660	2.3055		X1760_splice
MB-4674	-0.4127		G1087E
MB-5323	1.12		E880Rfs*13
MB-5328	-0.4891	C670W	
MB-5329	0.9207	V3365_S3366insELY,S3366Mfs*2	
MB-5377	-0.8126	F506L	
MB-4828	-0.6695		C305S
MB-5428	-1.0815		S1266T
MB-5465	2.8145		D560Efs*6
MB-5511	-0.5431	E2844K	
MB-5519	-0.2903	N319S	
MB-5635	-0.8715	S1115Lfs*4	
MB-6047	0.6405		E1287K
MB-6098	3.903		D1692N
MB-6100	-1.0737	E1514K	
MB-5232	-0.0813		X1453_splice
MB-6178	-0.822		V452A
MB-5275	0.2694	G4V	
MB-6271	0.6019		L502Sfs*2
MB-7032	-0.5413		K1667Qfs*11
MB-7038	0.5571		Q202Kfs*32
MB-7040	-0.5078		R1074T
MB-7048	-0.0828		F709Sfs*29
MB-6248	0.0592		X1663_splice
MB-5554	-0.4871	I1583T	
MB-6167	-0.2555	D806N	

Breast Invasive carcinoma dataset of 825 tumors

Sample	CDC25A mRNA	BRCA2 Mutation	BRCA1 Mutation
TCGA-BH-A18Q-01	0.6267	N3124I	
TCGA-A2-A0T0-01	1.0833	L2926*,I2315Kfs*12	
TCGA-A2-A0D2-01	1.1739		V1713*
TCGA-AO-A124-01	1.5915	C3304S	E23Vfs*17
TCGA-B6-A0X1-01	0.8325		E23Sfs*8
TCGA-A1-A0SO-01	3.0895		X27_splice
TCGA-A8-A07R-01	1.4493	I1470Kfs*11	
TCGA-AN-A0AL-01	0.6914		Q1756Pfs*74
TCGA-AN-A0AT-01	3.1272	E2650Q	
TCGA-AN-A0FL-01	0.6886		E1346Kfs*20
TCGA-AN-A0FX-01	0.0202		Q1323*
TCGA-AN-A0XU-01	0.6788		G1788V
TCGA-AR-A0U4-01	0.1673		N1121Kfs*12
TCGA-AR-A1AI-01	1.1136	R2336C	
TCGA-BH-A0BL-01	0.6832		C61G
TCGA-BH-A0WA-01	1.0708		X1559_splice
TCGA-C8-A12K-01	0.7977		R1699W
TCGA-D8-A147-01	1.087		C61G
TCGA-E2-A14N-01	2.0749		E111Gfs*3
TCGA-A8-A08L-01	0.5118	X2602_splice	
TCGA-A8-A07I-01	0.6939	D1355Y	
TCGA-A8-A08B-01	0.0947	V1270del	
TCGA-C8-A12T-01	1.8259	E3177Q	D1344H
TCGA-BH-A0DS-01	-0.1028		Q1811L
TCGA-AO-A03V-01	-1.1661	S1982Rfs*22	
TCGA-BH-A0B4-01	-0.7578	L2736Pfs*28	
TCGA-A2-A0SU-01	-0.5467	Y3308*	
TCGA-B6-A0RG-01	-0.2274	N1784Hfs*2	
TCGA-A1-A0SH-01	-1.4328		C61G,Q934*
TCGA-A8-A09A-01	-0.2405	E1953*	
TCGA-BH-A0AZ-01	-0.9148	S1955*	
TCGA-E2-A14Z-01	0.1746		X198_splice
TCGA-A8-A085-01	0.199	E260Sfs*15	
TCGA-A8-A097-01	0.4433	V220Ifs*4	
TCGA-A8-A09W-01	1.4261	E1518*	

TCGA-BH-A0BZ-01	-0.2904	T3033Nfs*11	
TCGA-E2-A14W-01	-0.6481	S599*	
TCGA-AO-A03T-01	0.0655	D3073G	

Supplementary Table 1. BRCA1 and BRCA2 mutated tumor samples analyzed in Fig. 7B METABRIC dataset

Supplementary Table 2.

Figure 1F

	MCF7Neo-NS vs. MCF7BRE-NS	MCF7Neo vs. MCF7Neo-BRCA2 shRNA#1	MCF7Neo vs. MCF7Neo-BRCA2 shRNA#2	MCF7Neo-BRCA2 shRNA#1 vs. MCF7BRE-BRCA2 shRNA#1	MCF7Neo-BRCA2 shRNA#2 vs. MCF7BRE-BRCA2 shRNA#2
**Day 5**	0.5778	0.015	0.005	0.0401	0.0149
**Day 7**	0.3231	0.0004	0.001	0.0341	0.046
**Day 9**	0.1347	<0.0001	<0.0001	0.0085	0.0403

Figure 3A

Radiation Dose (Gy)	*Brca2*$^{KO/KO}$*;Clone 3d*	*Brca2*$^{KO/KO}$*; MSCV-BRE*	*Brca2*$^{CKO/KO}$*;MSCV-BRE*		
5	0.0014	0.0027	0.0002		
10	0.0362	<0.0001	0.0014		
20	0.0005	0.0021	0.0001		

Figure 3B (CDC25A)

	Brca2$^{CKO/KO}$	*Brca2*$^{CKO/KO}$*;MSCV-BRE*			
**0.5 hr**	0.133	0.9431			
**1 hr**	0.0267	0.3267			
**2 hr**	0.0233	0.7956			

Figure 3B (p-CDK1)

	Brca2$^{CKO/KO}$	*Brca2*$^{CKO/KO}$*;MSCV-BRE*			
**0.5 hr**	0.0985	0.5977			
**1 hr**	0.0372	0.1552			
**2 hr**	0.0443	0.9062			

Figure 3C

	-BRE	+BRE			
**4 hr**	0.0015	0.5309			
**8 hr**	0.0242	0.6123			

Figure 3D

	BRE siRNA-NS	BRE siRNA #1	BRE siRNA #2		
**1 hr**	0.3686	0.0091	0.0089		
**2 hr**	0.18	0.0019	0.0009		
**3 hr**	0.0169	<0.0001	<0.0001		
**4 hr**	0.0052	0.0002	<0.0001		
**5 hr**	<0.0001	0.0002	<0.0001		
**6 hr**	<0.0001	<0.0001	0.0002		
**8 hr**	0.1013	0.0003	0.0002		

Figure 3E

MERIT40 siRNA	NS	NS	#1	#2	
**BRE**	-	+	+	+	
**4 hr**	0.0062	0.7065	0.0379	0.035	
**8 hr**	0.7383	0.7281	0.4568	0.4111	

Figure 5A

	$Brca2^{CKO/KO}$	$Brca2^{CKO/KO}$;MSCV-BRE	$Brca2^{CKO/KO}$;MSCV-BREΔ123		
**0.5 hr**	0.1416	0.9312	0.1145		
**1 hr**	0.0143	0.3792	0.0301		
**2 hr**	0.0346	0.5809	0.1148		

Supplementary Figure 2B

Olaparib concentration (μM)	$Brca2^{KO/KO}$;MSCV-BRE#1	$Brca2^{KO/KO}$;MSCV-BRE#2			
**0.001**	0.2624	0.1033			
**0.01**	<0.0001	0.0002			
**0.1**	<0.0001	0.0004			
**1**	0.0172	0.007			
**10**	0.6462	0.562			

Supplementary Figure 2C

Cisplatin concentration (µM)	*Brca2$^{KO/KO}$;MSCV-BRE#1*	*Brca2$^{KO/KO}$; MSCV-BRE#2*			
**0.2**	0.3676	0.2556			
**0.4**	0.0041	0.0024			
**0.6**	0.0006	0.0008			
**1**	0.003	0.001			
**1.2**	0.0066	0.0006			
**1.5**	0.3595	0.2506			

Supplementary Figure 2D

IR dose (Gy)	*Brca2$^{KO/KO}$;MSCV-BRE#1*	*Brca2$^{KO/KO}$; MSCV-BRE#2*			
**0.5**	0.0943	0.043			
**1**	0.0006	0.0006			
**2**	0.0006	0.0012			
**4**	<0.0001	0.0014			
**6**	0.7996	0.0114			

Supplementary Figure 3A

	Brca2$^{CKO/KO}$	*Brca2$^{KO/KO}$; Clone 3d*			
**0.5 hr**	0.0263	0.1331			
**1 hr**	0.0067	0.2167			
**2 hr**	0.0243	0.453			

Supplementary Figure 3B

	Brca2$^{CKO/KO}$	*Brca2$^{KO/KO}$; MSCV-BRE*			
**0.5 hr**	0.0214	0.0737			
**1 hr**	0.0082	0.431			
**2 hr**	0.0022	0.1726			

Supplementary Figure 4A

	NS	siRNA-A	siRNA-B	siRNA-C	
**8 hr**	0.2017	0.006	0.0031	0.0035	

Supplementary Figure 4B

	NS	siRNA-A	siRNA-B	siRNA-C	
**8 hr**	0.8665	0.0074	0.0054	0.0056	

Supplementary Figure 4C

	NS	siRNA-A	siRNA-B	siRNA-C	
**8 hr**	0.4271	0.0426	0.044	0.3667	

Supplementary Figure 5C

**15 minutes**	0.0453				
**30 minutes**	0.0008				
**60 minutes**	0.0365				
**120 minutes**	0.0253				

Supplementary Figure 11 H

**Olaparib concentration (µM)**	***Brca2^{KO/KO};MSCV-Cdc25a#1***	***Brca2^{KO/KO};MSCV-Cdc25a#2***			
**0.001**	0.0008	0.4037			
**0.01**	0.0003	<0.0001			
**0.1**	0.0001	0.0001			
**1**	0.0044	0.002			
**10**	0.9392	0.151			

Supplementary Figure 11 I

**Cisplatin concentration (µM)**	***Brca2^{KO/KO};MSCV-Cdc25a#1***	***Brca2^{KO/KO};MSCV-Cdc25a#2***			
**0.2**	0.9832	0.4646			
**0.4**	0.0003	0.0003			
**0.6**	0.0001	0.0004			
**1**	0.0048	0.0086			
**1.2**	0.0114	0.0048			
**1.5**	0.0075	0.0076			

Supplementary Figure 11 J

IR dose (Gy)	*Brca2$^{KO/KO}$;MSCV-Cdc25a#1*	*Brca2$^{KO/KO}$; MSCV-Cdc25a#2*			
**0.5**	0.0116	0.0103			
**1**	0.0012	0.0009			
**2**	0.0059	0.0003			
**4**	0.0494	0.0239			
**6**	0.3262	0.2794			

Supplementary Table 2. P values of statistical analysis of experiments shown in different figures. Figure numbers are mentioned on top of the each table

References

1. Chang, S., Biswas, K., Martin, B.K., Stauffer, S. & Sharan, S.K. Expression of human BRCA1 variants in mouse ES cells allows functional analysis of BRCA1 mutations. *The Journal of clinical investigation* **119**, 3160-3171 (2009).
2. Kuznetsov, S.G., Liu, P. & Sharan, S.K. Mouse embryonic stem cell-based functional assay to evaluate mutations in BRCA2. *Nature medicine* **14**, 875-881 (2008).
3. Biswas, K.*, et al.* A comprehensive functional characterization of BRCA2 variants associated with Fanconi anemia using mouse ES cell-based assay. *Blood* **118**, 2430-2442 (2011).
